# Supplementary figures and images for: The SUMO ligase MMS21 profoundly influences maize development through its impact on genome activity and stability
Source: PLoS Genet. 2021 Oct 25;17(10):e1009830. doi: 10.1371/journal.pgen.1009830 (PMC8568144; doi:10.1371/journal.pgen.1009830)

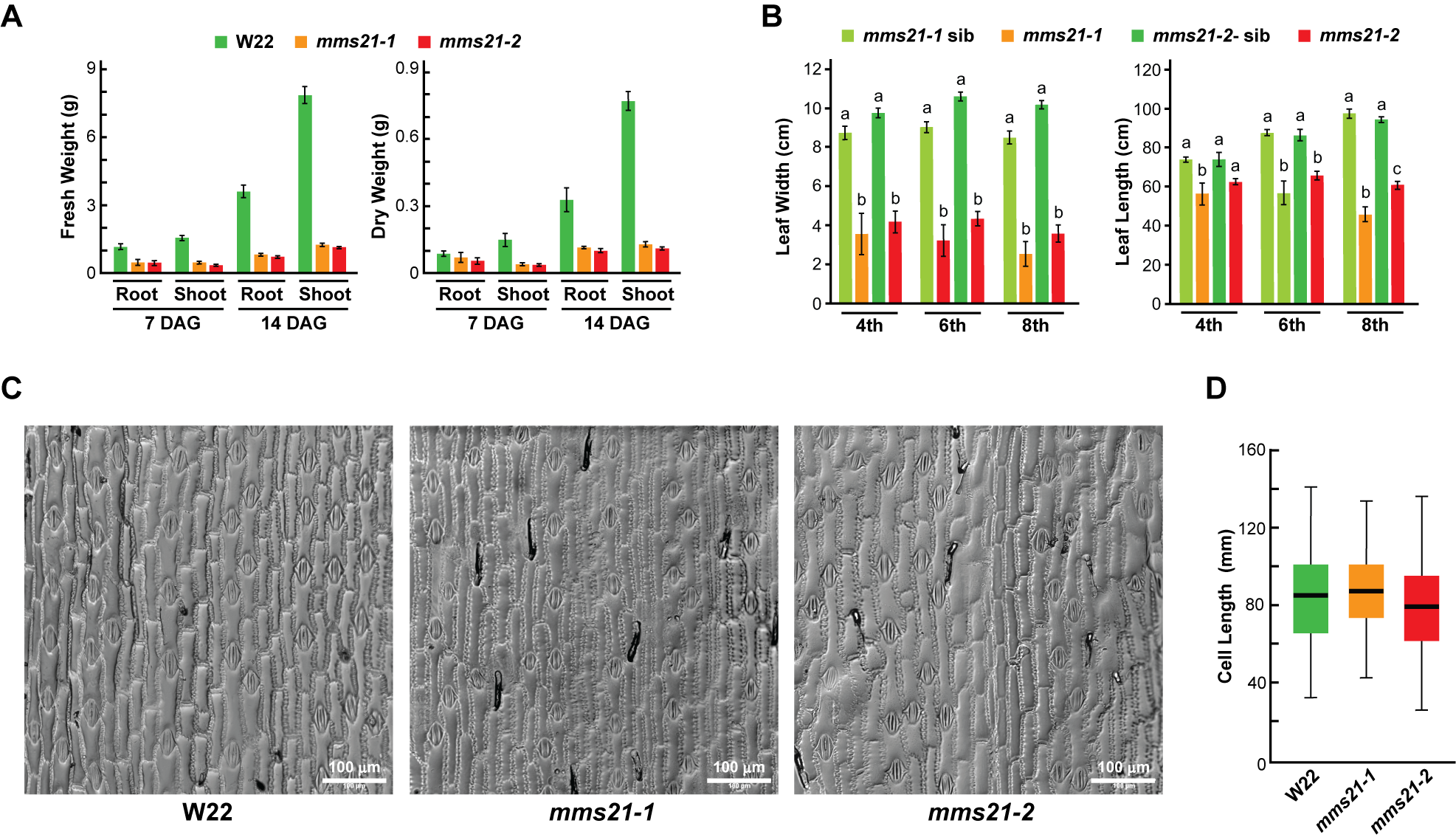

Supplement: S1 Fig — Shown are measurements of tissues from homozygous mms21-1, mms21-2, and W22 plants. (A) Quantification of fresh weight, dry weight, and root area of plants harvested at 7- and 14 (Days After Germination) DAG. Each bar represents the mean of 3 biological replicates, each with at least 3 plants (±SE). (B) Quantification of leaf length and width for the 4th, 6th, and 8th leaves (counted from the tassel) at maturity. Each bar represents the mean of 3 biological replicates, each with at least 3 plants (±SE). The a, b, and c notations identify values that were significantly different from one another and the normal sibling control, as determined by one-way ANOVA followed by the Tukey’s post hoc test (p-value <0.05). (C) Representative epidermal surface impressions of leaves. Micrographs of glue slide impressions were prepared from abaxial surface of mature leaves adjacent to the tassel. Bars = 100 μm. (D) Box plots of cell lengths for leaves grown as in panel (C). Cell lengths were measured using ImageJ and plotted in R (n = 200). (TIF) [file pgen.1009830.s001.tif]

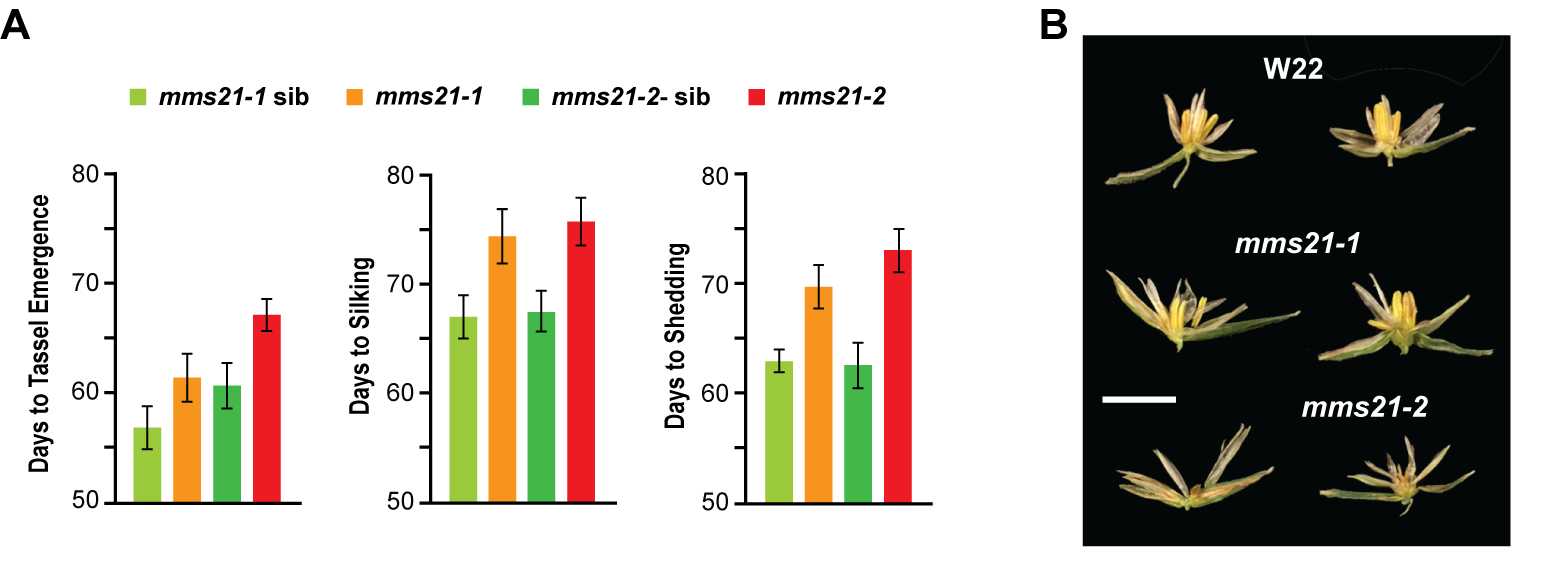

Supplement: S2 Fig — (A) Quantification of defects seen for mms21-1 and mms21-2 anther development as compared to those from normal siblings. Traits measured include: days to tassel emergence, days to silking, and days to pollen shedding. Each bar represents the average of three biological replicates (±SD). (B) Dissection of male florets showing that male floral morphology is relatively normal for mms21-1 but abnormal for mms21-2. Scale bar = 8 mm. (TIF) [file pgen.1009830.s002.tif]

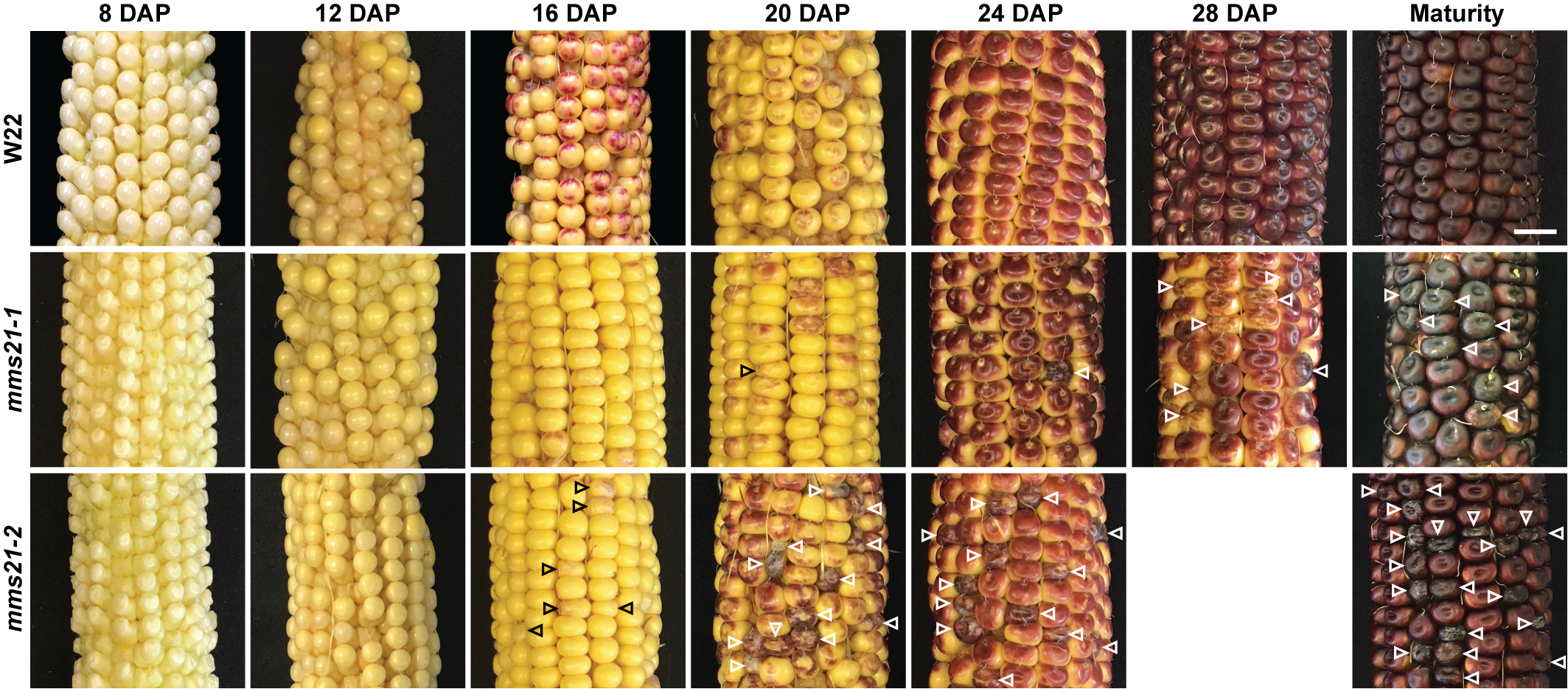

Supplement: S3 Fig — Shown are cobs from self-pollinated W22, and mms21-1/+, and mms21-2/+ plants from 8 DAP to maturity. Arrowheads locate obvious defective kernels. (TIF) [file pgen.1009830.s003.tif]

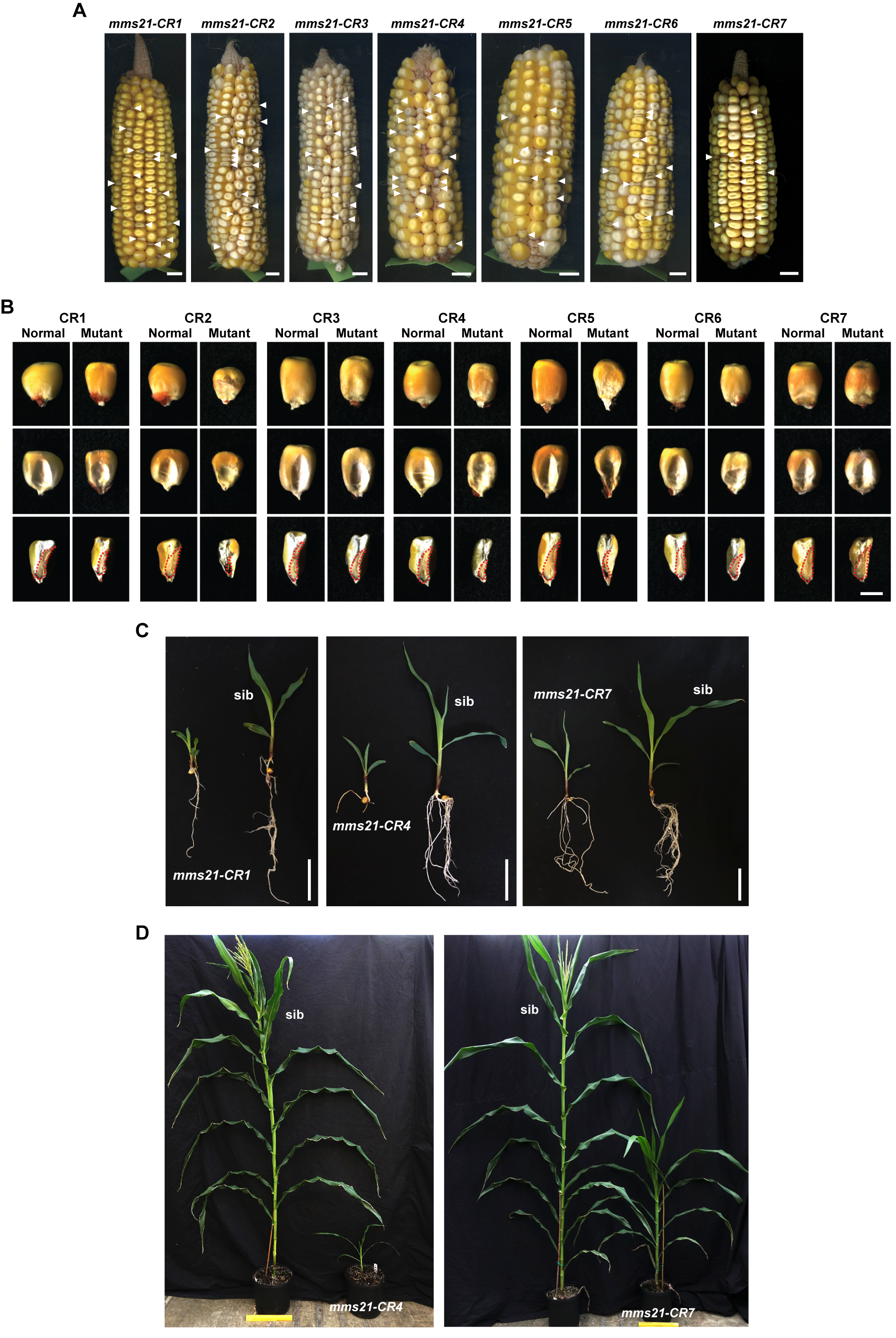

Supplement: S4 Fig — (A) Mature ears from self-pollinated heterozygous mms21 CRISPR/Cas9 (CR) alleles showing the appearance of defective kernels (arrowheads). Scale bars = 1 cm. (B) Abnormal morphology of mms21-CR seeds. The abgerminal (top), germinal sides (middle) and the saggital sections (bottom) of one representative seed is shown. Scale bar = 5 mm. (C) Homozygous mms21-CR mutants imaged 2-week after planting. Scale bars = 5 cm. (D) mms21-CR4 and mms21-CR7 plants imaged at flowering. Scale bars = 30 cm. Each seed/plant was compared to its phenotypically normal sibling. (TIF) [file pgen.1009830.s004.tif]

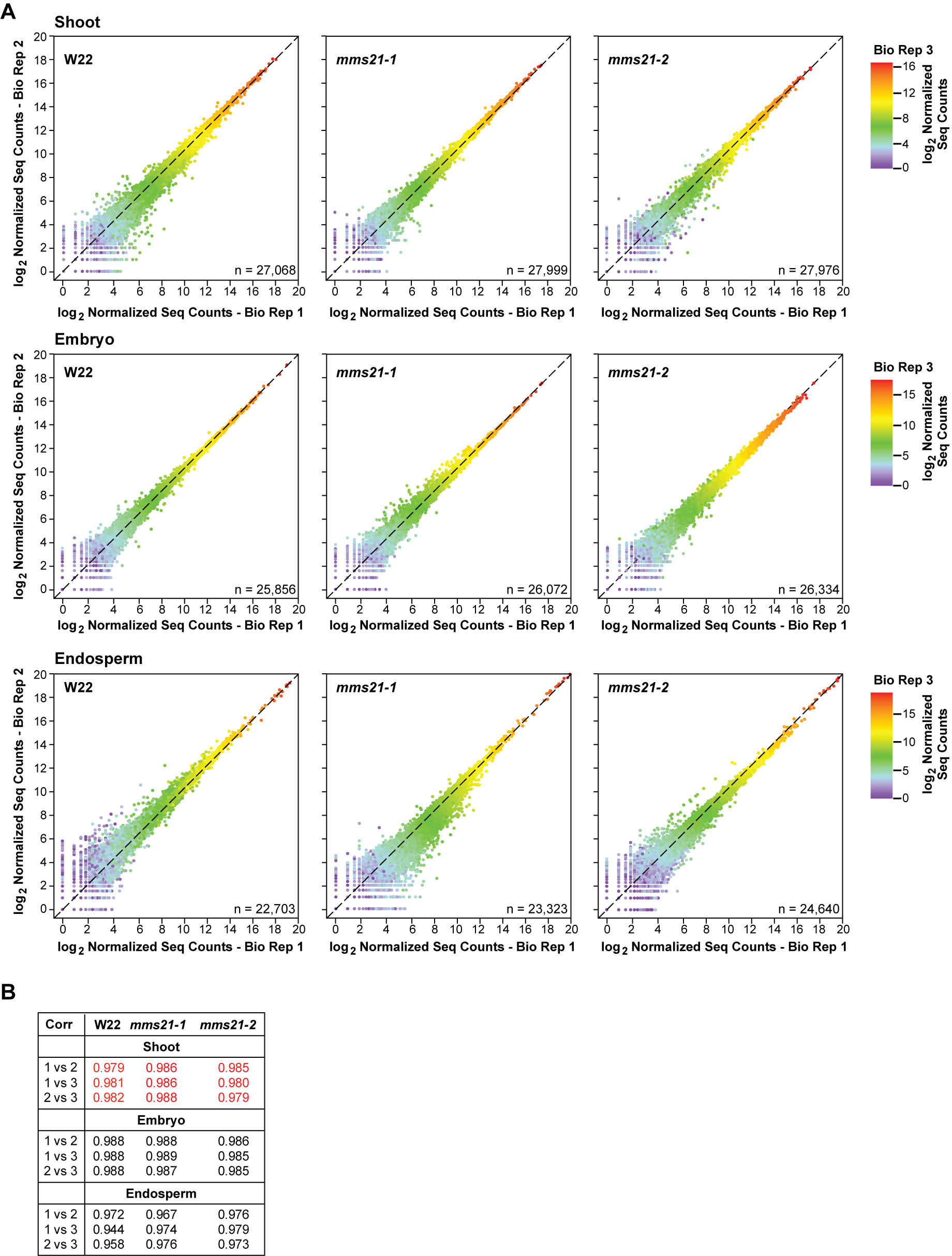

Supplement: S5 Fig — (A) The scatter plots comparing the transcript profiles obtained from three biological replicates from either W22, mms21-1, or mms21-2 shoots, developing embryos, or endosperm. Transcript abundances acquired by RNA-seq were expressed as log2-transformed normalized values. The total number of transcripts analyzed is shown at the bottom right corner. The dashed lines show a correlation = 1, correlation of the first two biological replicates were plotted, while the log2-transformed transcript abundances for the third biological replicate are superposed onto the scatter plot by the color gradient shown on the right. (B) The Pearson correlation coefficients among the samples for each genotype. (TIF) [file pgen.1009830.s005.tif]

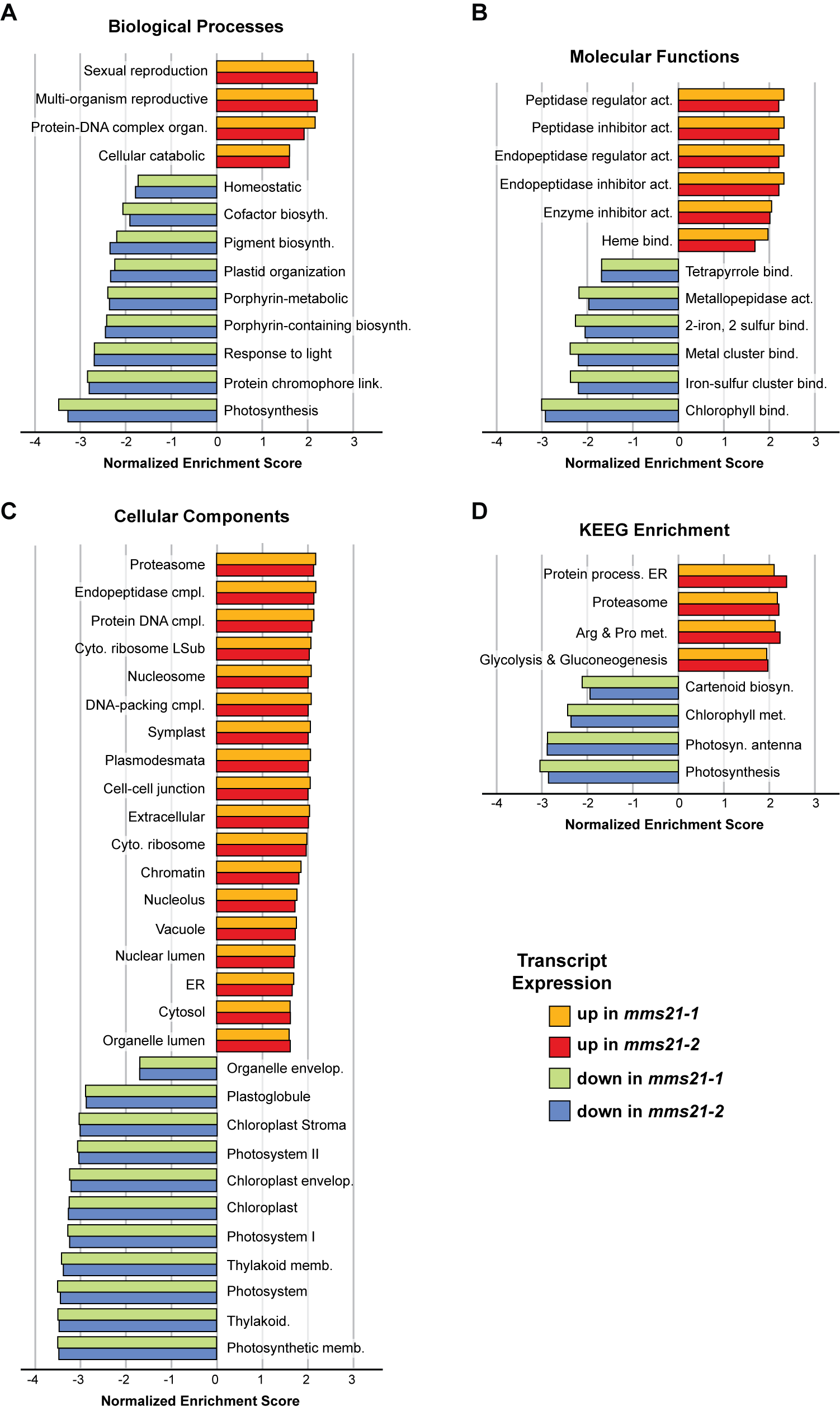

Supplement: S6 Fig — (A-C) GO term enrichment analysis of DEGs in the mms21-2 versus W22 shoots. The vertical coordinates indicate the enriched GO terms, and the horizontal coordinates show the normalized enriched score for each GO term. Negative values indicate downregulation, positive values indicate upregulation. GO enrichment was performed using all the three sub-ontologies: Biological Process (A), Molecular Function (B), and Cellular Component (C). (D) KEGG enrichment analysis of the DEGs. The vertical coordinates are the enriched pathways, and the horizontal coordinates are the normalized enriched score for each GO term. (TIF) [file pgen.1009830.s006.tif]

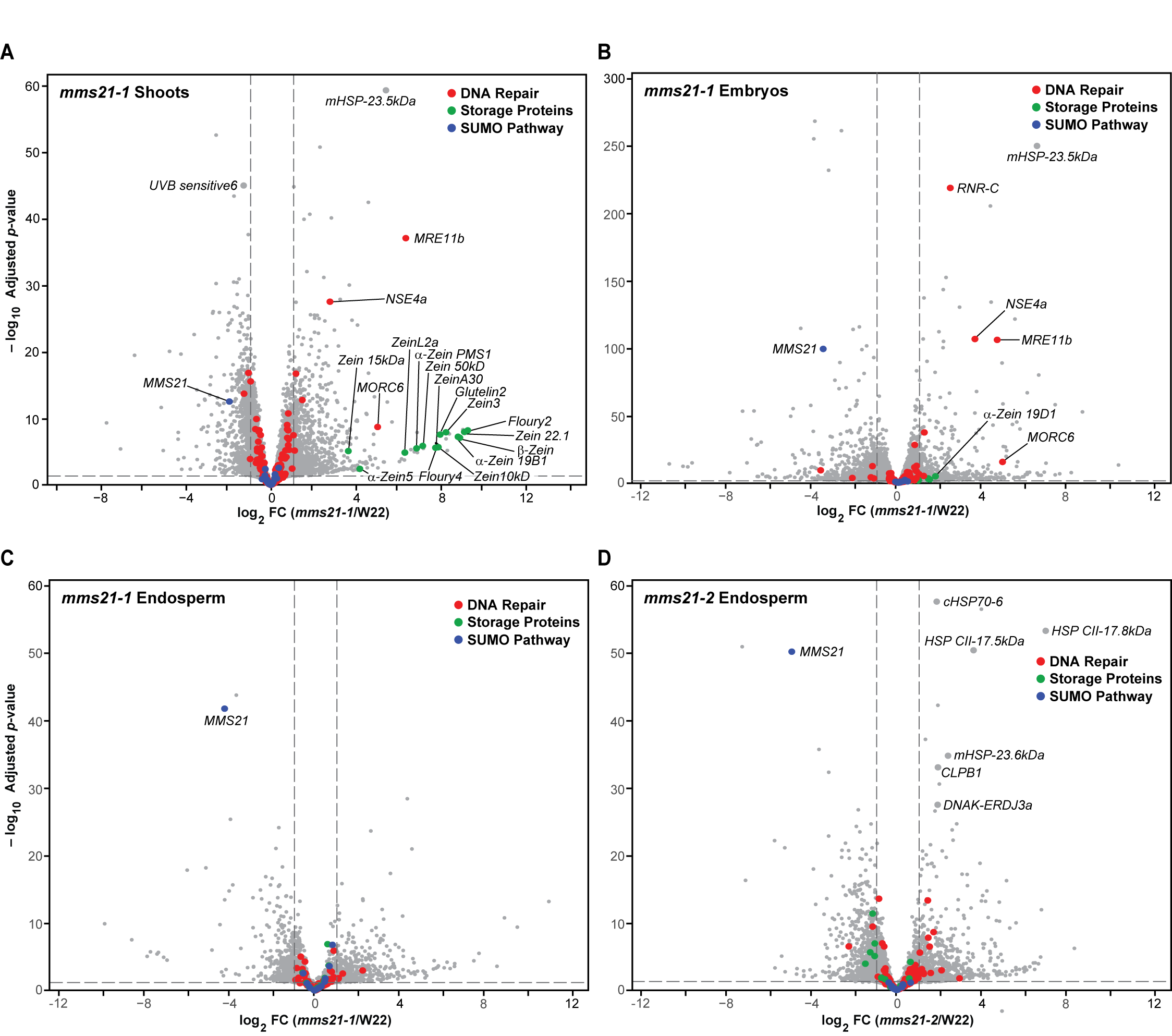

Supplement: S7 Fig — Volcano plot representation of DEGs in mms21 versus the W22 based on FC and -log10 adjusted p-values. Shown are transcriptome analyses of mms21-1 shoots (A), mms21-1 embryos (B), mms21-1 endosperm (C), and mms21-2 endosperm (D). Analysis of other tissues are shown in Fig 6A. The horizontal and vertical dashed lines indicate a FC = 2 and an adjusted p-value = 0.05. Transcripts encoding proteins involved in DNA repair, maize zein storage proteins, and the SUMO pathway are indicated in red, green and blue points, respectively. All other transcripts are shown in grey. Specific mRNAs of interest that were significantly altered in expression are noted. (TIF) [file pgen.1009830.s007.tif]

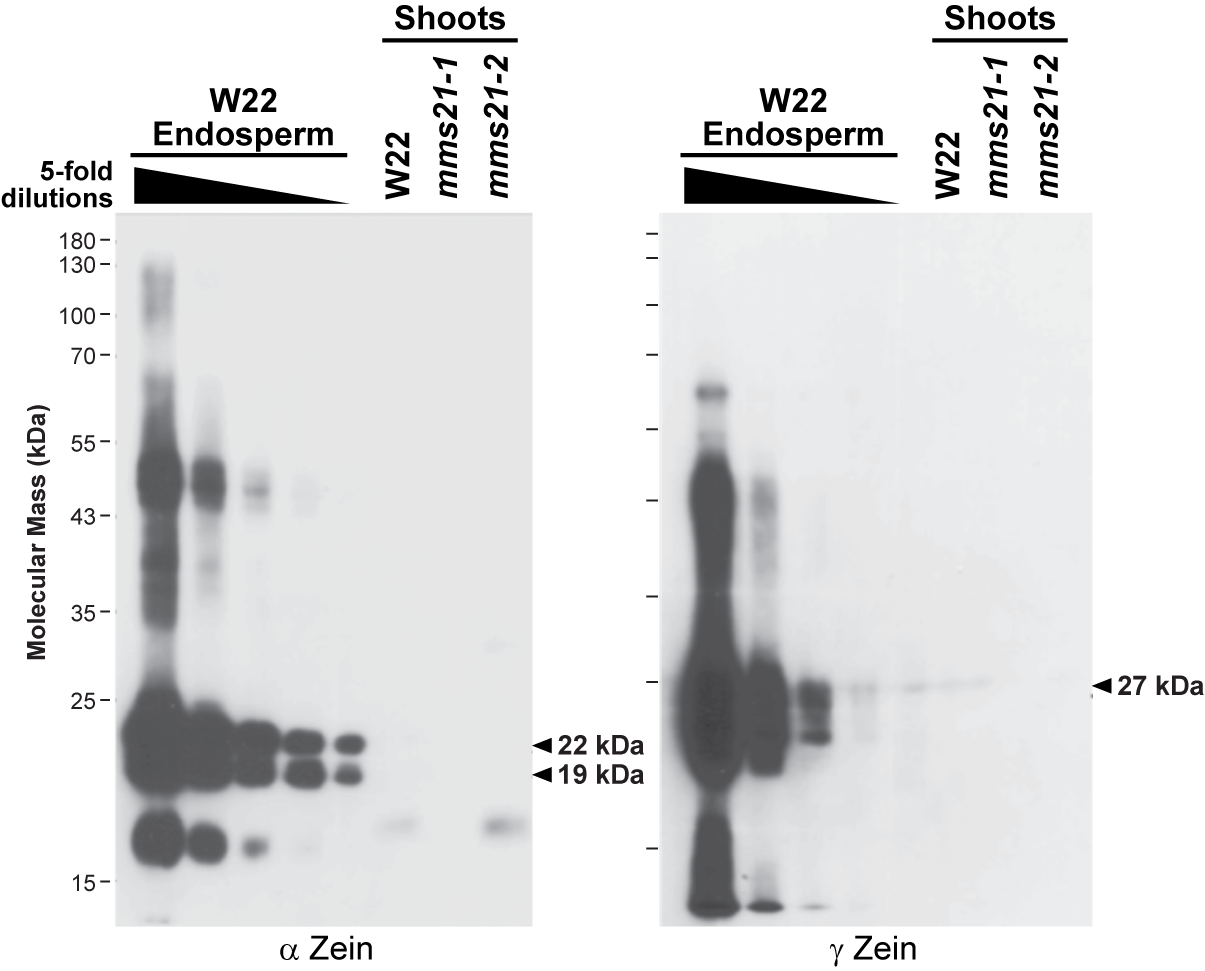

Supplement: S8 Fig — Ethanol soluble proteins from mms21-1, mms21-2, and W22 shoots harvested at 10 DAS were subjected to SDS-PAGE and immunoblot analysis with antibodies against the 19- and 22-kDa α-zeins, or the 27-kDa γ-zeins. Five-fold serial dilutions after a 10-fold dilution of W22 endosperm dissected from dry seeds are shown in the left lanes for comparisons. Seedling samples were loaded on equal dry weight basis. The migration positions of the zeins are indicated by the arrowheads. (TIF) [file pgen.1009830.s008.tif]

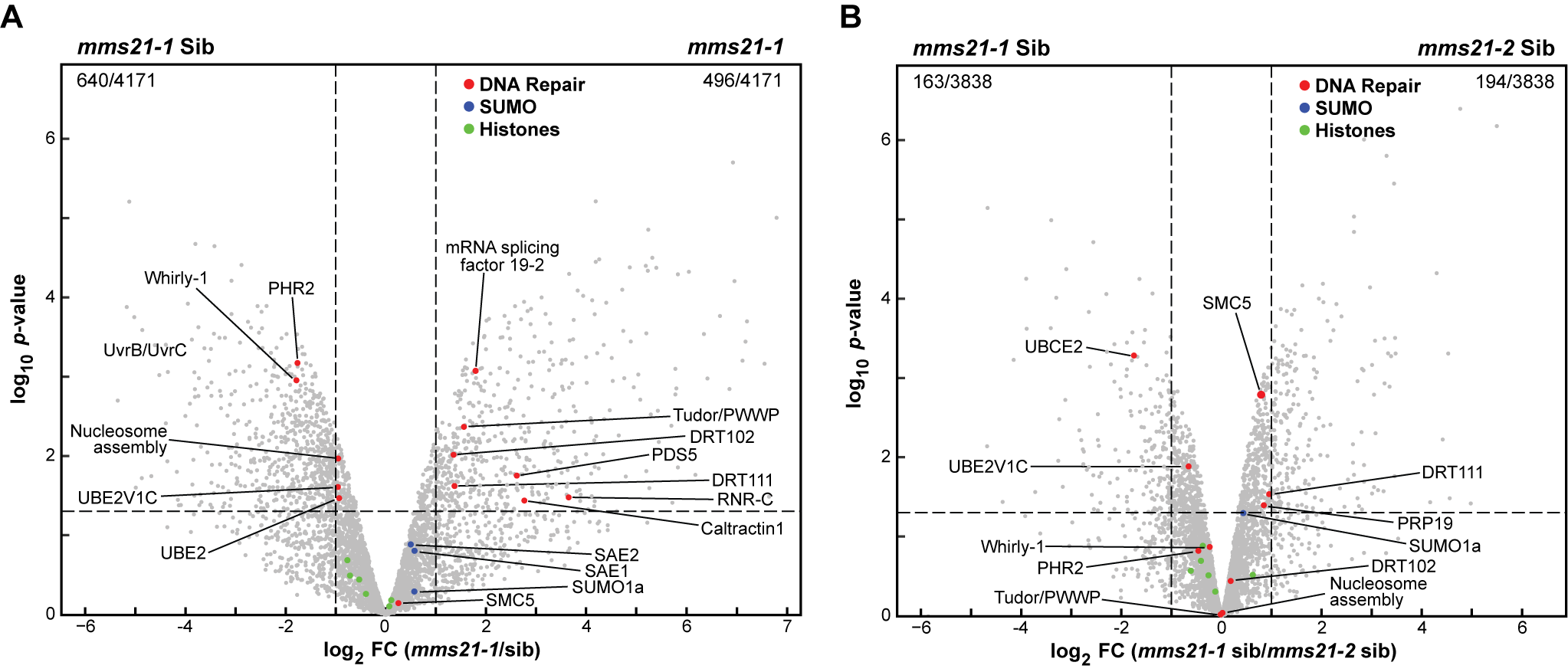

Supplement: S9 Fig — (A) Altered proteome profile for the mms21-1 mutant. The volcano plot depicts protein abundance changes for 4,171 proteins detected by MS from mms21-2 leaves as compared to those of its normal sibling. (B) Proteome profile comparison of leaves from mms21-1 normal siblings and mms21-2 normal siblings. Each dot represents one protein that had detectable expression in both samples and was plotted based on its log2 FC in abundance (mutant/normal siblings) and its -log10 p-value of significance based on the three biological replicates, each with two technical replicates. The horizontal and vertical dashed lines mark a FC = 2 in protein abundance and a p-value = 0.05, respectively. Histone proteins used to confirm data normalization are shown as green. SUMO pathway components and DNA repair-associated proteins are highlight in blue and red, respectively. (TIF) [file pgen.1009830.s009.tif]

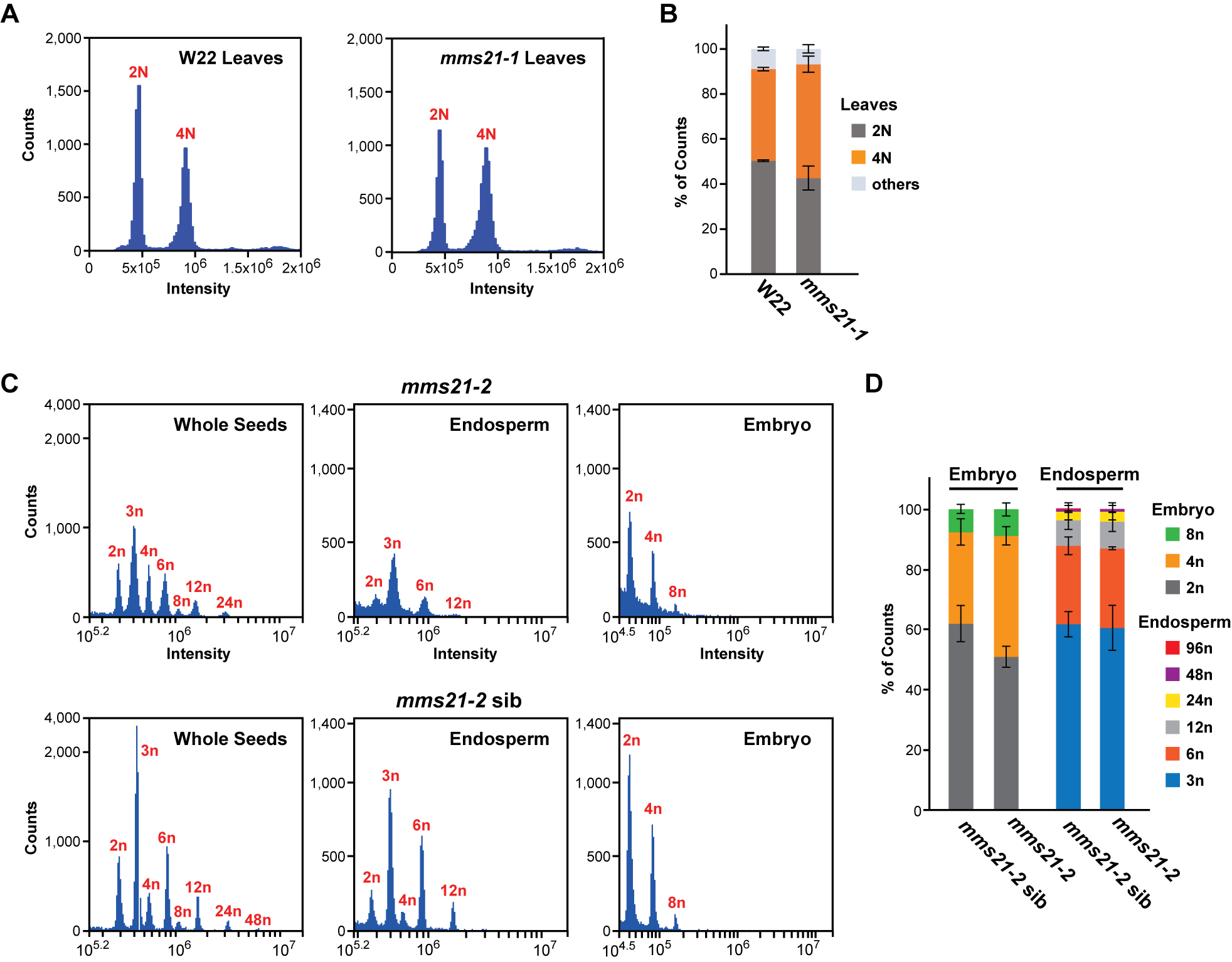

Supplement: S10 Fig — Nuclei were isolated from seedling leaves, whole seeds, endosperm and embryos, stained with propidium iodine, and subjected to FACS sorting to determine ploidy number based on fluorescence counts. (A) Distribution of nuclei ploidy levels in seedling leaves from 10 DAS plants (2n and 4n). (B) Quantification of ploidy levels derived from seedling leaf nuclei analyzed in panel (A). Each bar represents the percent of the respective average totals obtained from three biological replicates, each of which was prepared from one seedling (±SD). (C) Distribution of nuclei at various ploidy levels (2n to 96n) in whole seeds, and dissected endosperm and embryos at 16 DAP. The predicted ploidy levels of the various fluorescence peaks are indicated. (D) Quantification of ploidy levels embryo and endosperm nuclei analyzed in panel (C). Each bar represents the percent of the respective average totals obtained from three biological replicates, each of which was prepared from three pooled seeds (±SD). (TIF) [file pgen.1009830.s010.tif]

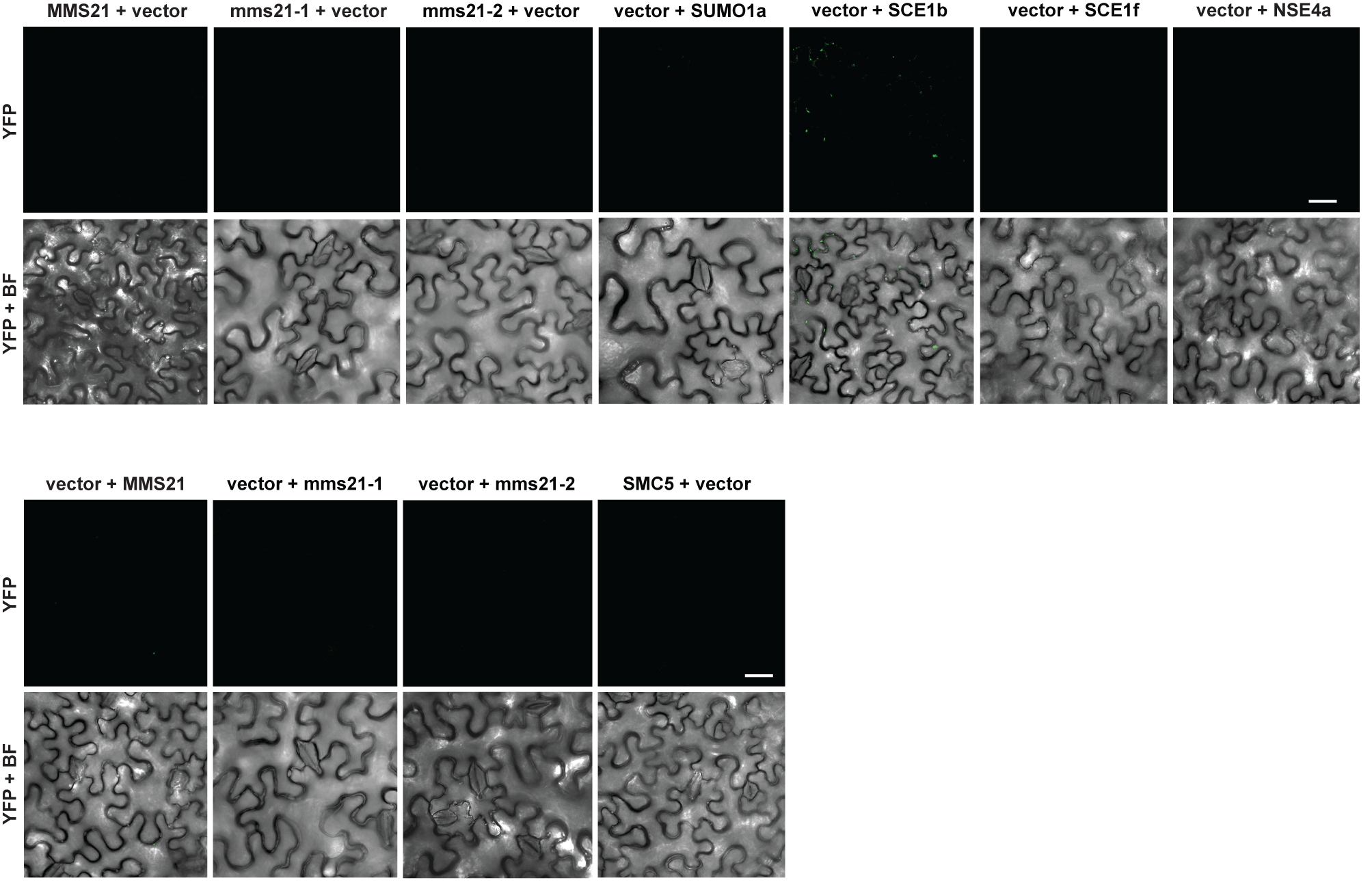

Supplement: S11 Fig — Pairwise expression of MMS21 and its potential interactors fused to the N-terminal (nYFP) or C-terminal (cYFP) halves together with the nYFP and cYFP fragments by themselves. N. benthamiana leaf epidermal cells were co-infiltrated with the indicated plasmid combinations, and fluorescence signals were detected by confocal fluorescence microscopy 40–45 h after infiltration. Shown are the fluorescence images alone or merged with their companion bright field images. Only the cYFP-SCE1b construction expressed by itself generated a subtle fluorescence signal due to auto-activation. Scale bars = 40 μm. (TIF) [file pgen.1009830.s011.tif]

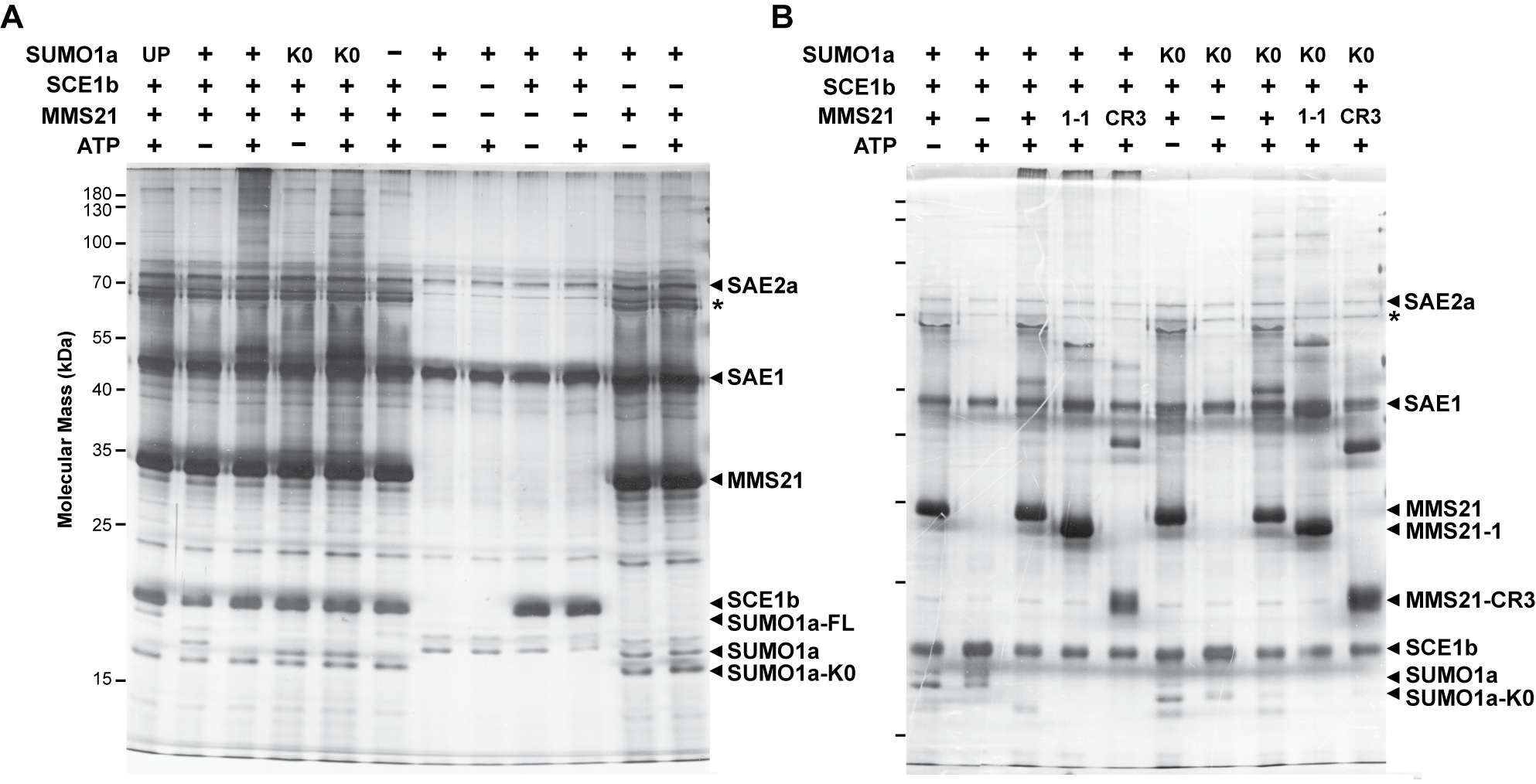

Supplement: S12 Fig — The SUMOylation reaction mixtures are identical to those described in Fig 10A and 10B. The mixtures were subjected to SDS-PAGE and stained for protein with silver. The migration position for each component is indicated by the arrowheads. An unknown contaminant is highlighted by the asterisk. (TIF) [file pgen.1009830.s012.tif]

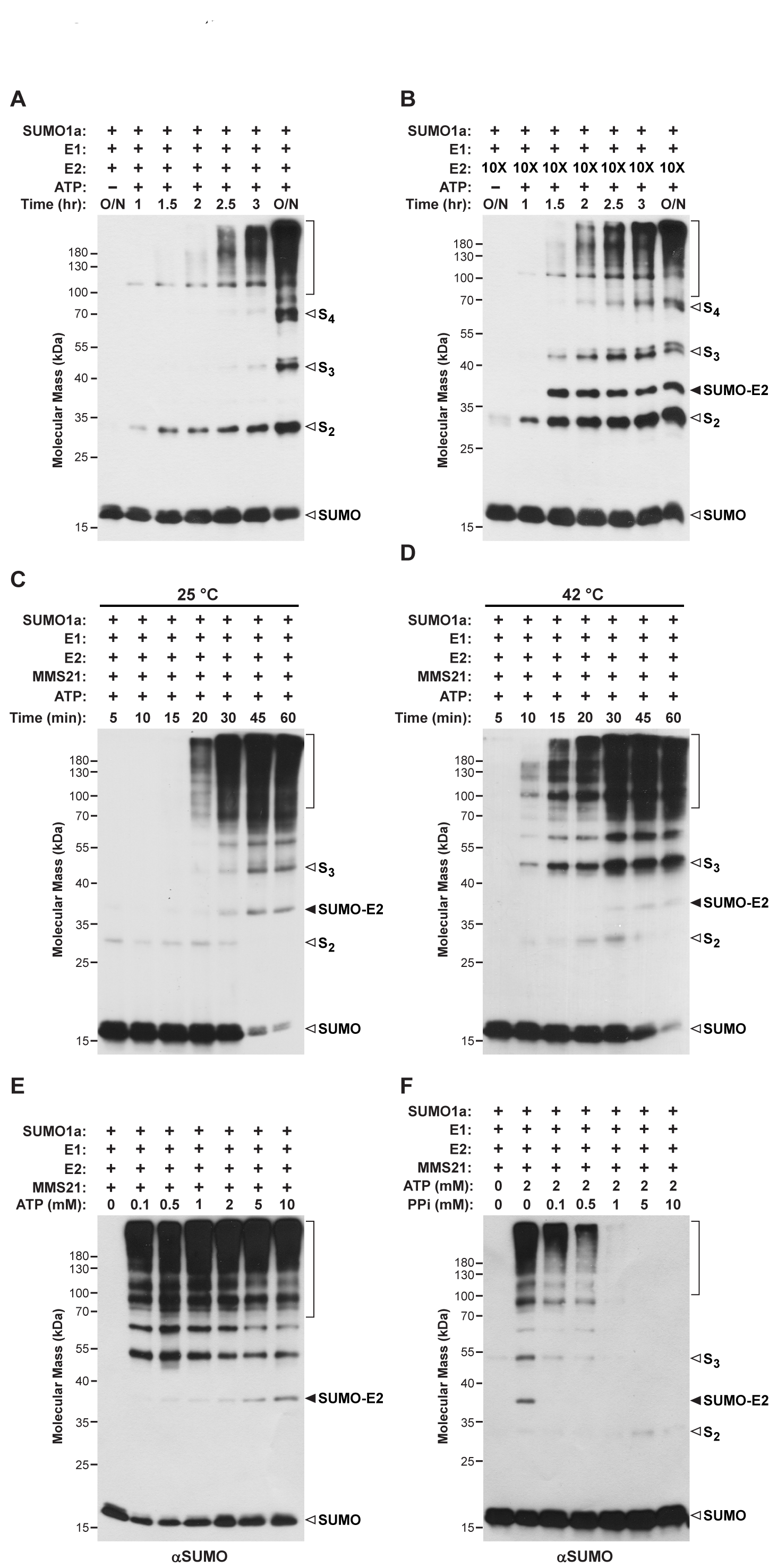

Supplement: S13 Fig — Recombinant versions of full-length SUMO E1 (SAE1/SAE2), the SUMO E2 SCE1b, the processed and active version of SUMO1a, and full-length MMS21 were affinity purified and mixed in various combinations with or without ATP. After quenching the reactions with SDS-PAGE sample buffer, the mixtures were subjected to SDS-PAGE and immunoblot analysis with anti-SUMO1 antibodies. (A) Time course of SUMO conjugation for reactions containing only SUMO1a, the SAE1/SAE2 E1 heterodimer, the SCE1b E2, and ATP. O/N, overnight. (B) Time course of SUMO conjugation for reactions as panel (A) but with 10 times more of SCE1b E2 enzyme. O/N, overnight. (C) Time course for MMS21-directed SUMO conjugation at 25°C for reactions containing SUMO1a, the SAE1/SAE2 E1 heterodimer, the SCE1b E2, and ATP. (D) Time course for MMS21-directed SUMO conjugation at 42°C as in panel (C). (E) Complete SUMO conjugation reactions containing SUMO1a, the SAE1/SAE2 E1 heterodimer, the SCE1b E2, MMS21 E3, and various concentrations of ATP. (F) Pyrophosphate (PPi) inhibits SUMOylation. MMS21-directed SUMO conjugation was conducted in the presence of increasing concentration of PPi in reactions containing SUMO1a, the SAE1/SAE2 E1 heterodimer, the SCE1b E2, and 2 mM ATP. Unless indicated otherwise, the assays were performed at 25°C in 20 μL reaction volumes containing 4 μg of SUMO1a, 500 ng of SAE1 and 200 ng of SAE2a (E1), 400 ng of SCE1b (E2), and 1.3 μg of MMS21(E3), with or without 2 mM ATP. The reactions in panels (E) and (F) were performed for 1.5 hr. (TIF) [file pgen.1009830.s013.tif]

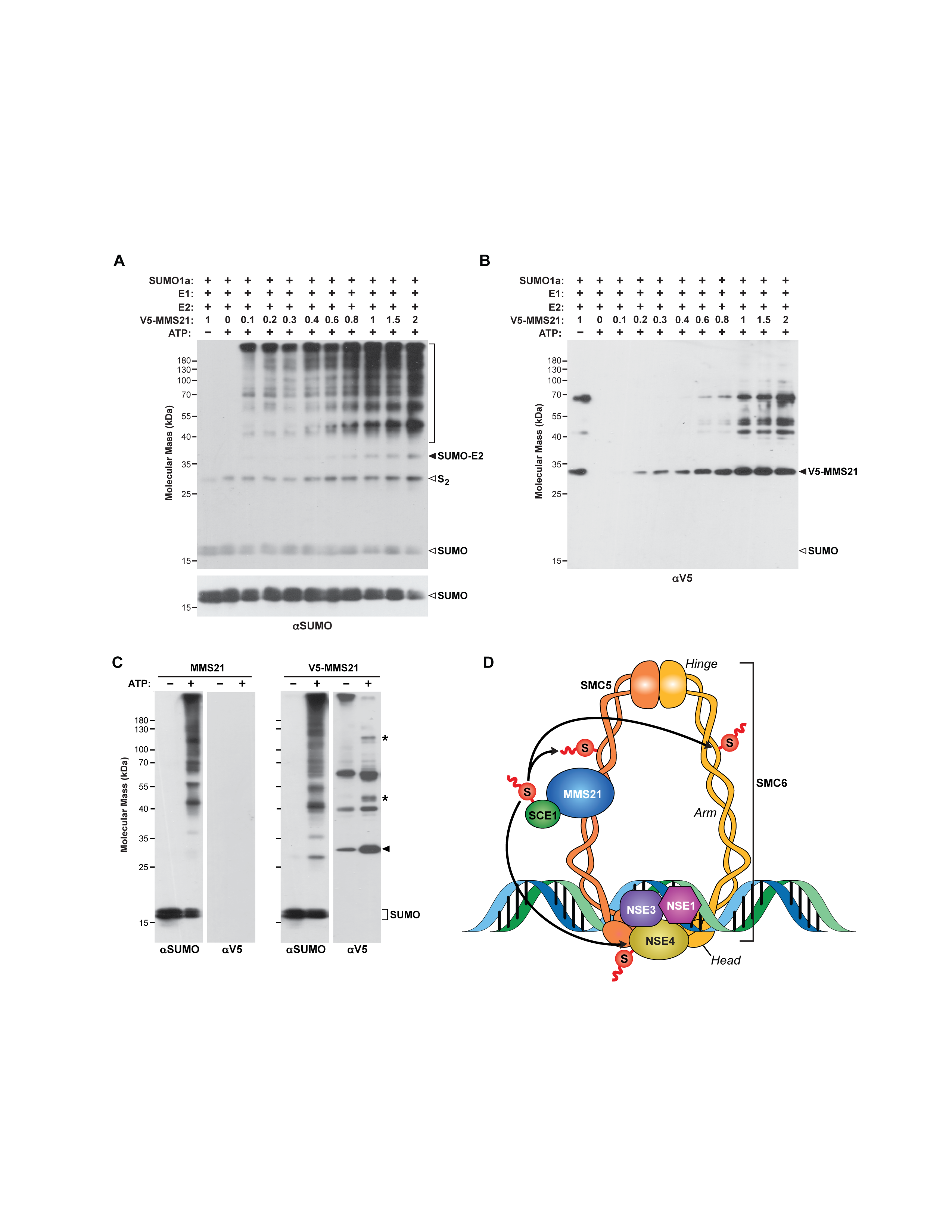

Supplement: S14 Fig — (A and B) in vitro reactions showing that MMS21 mainly SUMOylates other proteins within the reaction mixtures and not only MMS21 itself. Shown are in vitro SUMOylation in complete reaction mixtures containing increasing concentrations of V5-tagged MMS21 along with processed and active SUMO1a, the SAE1/SAE2 E1 heterodimer, the SCE1b E2, and 5 mM ATP. Reactions were conducted at 25°C for 1.5 hr in 20 μL volumes containing 4 μg of SUMO1a, 500 ng of SAE1 and 200 ng of SAE2a (E1), 400 ng of SCE1b (E2), and various amounts of MMS21 (E3), with or without 2 mM ATP as indicated. One part V5-MMS21 equals 1.3 μg. The products were subjected to SDS-PAGE and immunoblot analysis with anti-SUMO1 (A) or anti-V5 antibodies (B). (C) The N-terminal V5 tag did not impact the SUMO ligase activity of MMS21. Shown are complete SUMO conjugation reactions as in (A) containing 1.3 μg MMS21 expressed with (right panel) or without (left panel) the V5 tag. The arrowhead and bracket locate V5-MMS21 and free SUMO1a, respectively. The asterisks indicate SUMOylated forms of V5-MMS21 generated during the reaction. Note that the profile of conjugates detected with anti-SUMO1 antibodies differ markedly from that detected with anti-V5 antibodies. (D) Cartoon of the predicted three-dimensional structure of the SMC5/6 complex bound to DNA and containing its accessory factors NSE1, NSE3, and MMS21 (also known as NSE2). The Hinge, coiled-coil Arm, and Head domains of SMC5 and 6 are indicated. The SMC5/6 complex proteins known to be SUMOylated are shown. Adapted from [47]. S, SUMO. (TIF) [file pgen.1009830.s014.tif]

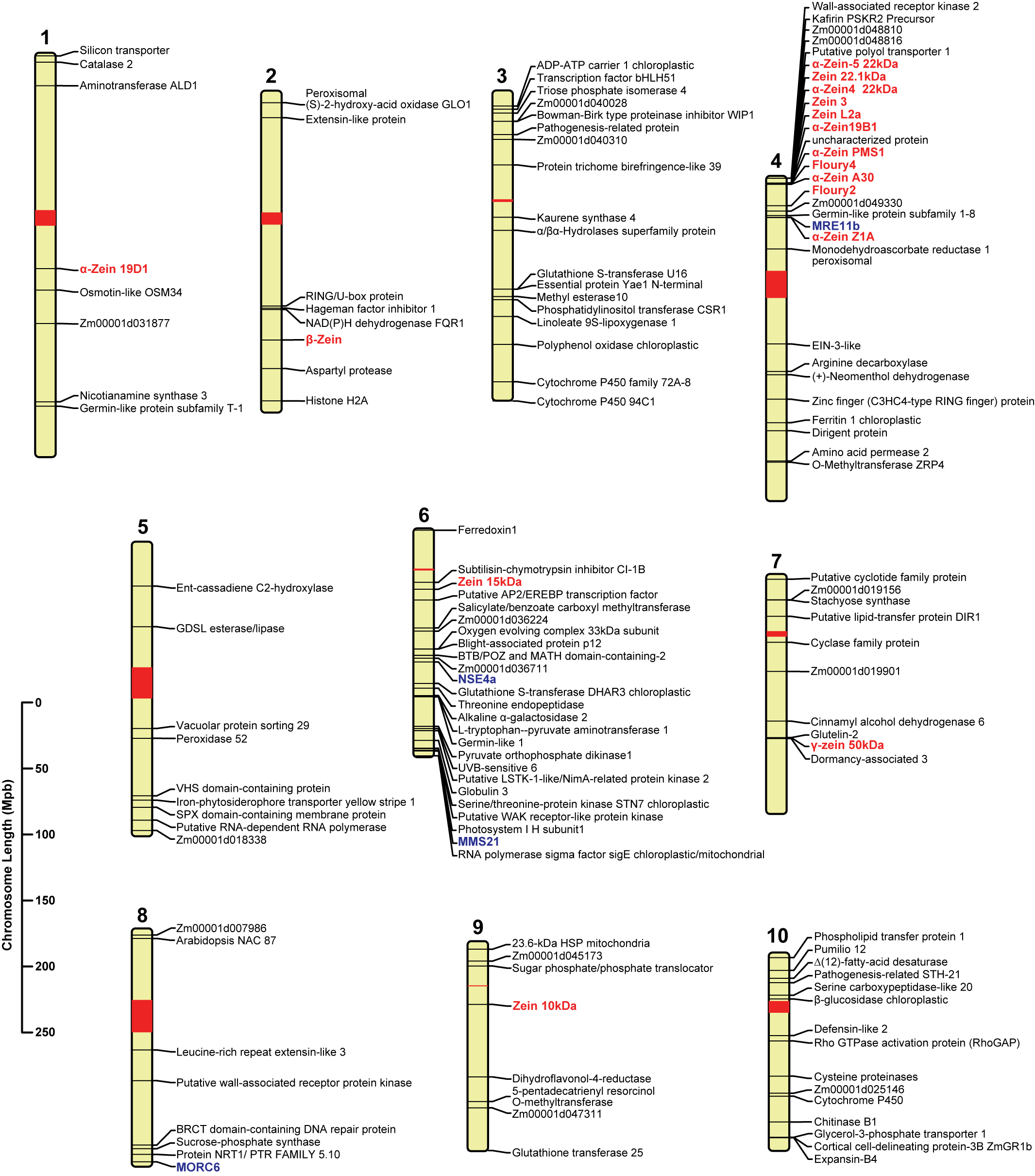

Supplement: S15 Fig — Positions within the 10 maize chromosomes were mapped by TBtools [80] for the collection of 146 DEG showing a FC >16 or <-16 or a -log10 adjusted p-value >20 (adjusted p-value <1e-20), either up or down. Centromeres are shown in red. Genes encoding zeins are highlighted in red while those for MMS21, NSE4a, MOR6, and MRE11b are highlighted in blue. (TIF) [file pgen.1009830.s015.tif]
