# Supplementary material for: The SUMO ligase MMS21 profoundly influences maize development through its impact on genome activity and stability
Source: PLoS Genet. 2021 Oct 25;17(10):e1009830. doi: 10.1371/journal.pgen.1009830 (PMC8568144; doi:10.1371/journal.pgen.1009830)
Supplement: Attachment — Submitted filename: MMS21 PLOS Genetics revision Cover Letter.doc [file pgen.1009830.s019.doc]

# DEPARTMENT OF BIOLOGY

# Richard D Vierstra, Ph.D.

# *George and Charmaine Mallinckrodt Professor of Biology*

Editorial Board

*PLOS Genetics*

*1265 Battery Street, Suite 200*

*San Francisco, CA 94111*

August 31, 2021

Dear Editorial Board,

Please find our revised draft of the manuscript titled “***The SUMO ligase MMS21 profoundly influences maize development through its impact on genome activity and stability***” by the co-first authors Drs. Junya Zhang and Robert Augustine along with other authors and myself for possible publication in *PLOS Genetics*. We appreciate the time and effort that the Reviewers dedicated to providing feedback on our manuscript and are grateful for the insightful comments and suggested improvements. Needless to say, we were pleased to read such positive comments on the work, and hope that this revised manuscript is now acceptable for publication.

The work describes in the revised manuscript reveals the potential functions of MMS21 using maize (*Zea mays*) as a genetic model that connect this SUMO ligase to seed, pollen germination and seed/vegetative development through controls on DNA-damage repair, chromatin stability and transcriptional control on proteome balance. Below please find a point-by-point response to the issues raised by the Reviewers, outlining how we have made the requested changes. As you will read, we feel that we have accommodated all the suggestions made by the reviewers. The main areas of the text modified to address the Reviewer’s comments are highlighted in red/yellow background in the revised marked-up version of the manuscript.

**Reviewer #1:**

*Introduction. The MMS21 protein is a known and evolutionary conserved subunit of the SMC5/6 complex. This is somehow lost in the introduction and I suggest making this point more prominent. Furthermore, it would be great mentioning that MMS21 / HPY2 is known also as NSE2.*

We thank the reviewer for this suggestion. We made changes to include NSE2 as another name for MMS21,and modified our introduction to make it clear that MMS21 is a conserved subunit of the SMC5/6 complex.

*The citations in the text often do not follow a chronological order. Please correct throughout the manuscript.*

We have reformatted our citation into a chronological order per the style of PLoS Genetics.

*The authors analyzed endoreduplication levels using seeds of mms21 plants and did not observe any difference to WT. Is it possible that the maize seeds already reached their maximum endoreduplication capacity and therefore do not show an enhanced phenotype in the mutants? All quantifications of endoreduplication levels in Arabidopsis (which the authors refer to) were done using somatic tissues. Therefore, I suggest repeating the experiment using leaf tissues*.

*The authors suggest defects in anther development, pollen germination and problematic seed development. This is reminiscent of a recently published results in Arabidopsis showing that mms21/nse2 mutants produce unreduced gametes and triploid progeny (Yang et al., Plant Cell, 2021). It would be great if the authors could analyze ploidy of the maize mms21 plants (in particular the poorly growing ones).*

We thank the reviewer for the suggestion and the additional reference. We agree that it is possible maize seeds have a limit on their endoreduplication capacity and it could be a reason for why we did not observe a difference of the ploidy level in the seeds. We performed a flow cytometry assay on the mutant seedling leaves as reviewer suggested, please find these data in Supplemental Figure 10. We still did not observe a high ploidy phenotype in maize *mms21* leaves, indicating that it is likely that the loss of MMS21 does not elicit a high ploidy phenotype in this plant species as seen in Arabidopsis.

*Line 522. I cannot fully agree with the sentence „Strikingly, while strong Arabidopsis MMS21 mutants are viable and fertile (Huang et al., 2009; Ishida et al., 2009), we found that the strongest maize alleles impacting MMS21 not only substantially attenuated fertility and seed formation, but also compromised vegetative development leading to progeny that either failed to germinate or arrested growth soon after germination“. Strong Arabidopsis mms21 mutants are also affected in their growth and fertility (e.g. Liu et al., BMC Plant Biol., 2014; Yang et al., Plant Cell, 2021). In fact, the phenotypes are very similar to what the authors describe for maize. Therefore, I suggest rewriting the sentence starting on line 522 and reformulating the sentence starting on line 526.*

We thank the reviewer for his/her insight. We have modified our discussion, please see line 514-517 in our revised manuscript.

**Reviewer #2**

Reviewer #2 did not have any specific concerns on our paper, and we thank this reviewer for his/her positive comments about the work.

We hope we have been able to fully address all the concerns of the Reviewers in a satisfactory manner and thank you in advance for your continued consideration of our manuscript. If you have any additional questions, please do not hesitate to contact us. We look forward to hearing from you.

Yours Sincerely,

Dr. Richard D. Vierstra

George and Charmaine Mallinckrodt Professor

Department of Biology, Campus Box 1137

Washington University in St. Louis

One Brookings Drive,

St. Louis, Missouri 63130 USA

(314)-935-5058 office;

(314)-935-4432 fax

[rdvierstra@wustl.edu](mailto:rdvierstra@wustl.edu)

https://biology.wustl.edu/people/richard-d-vierstra
